# Warburg effect regulated by amphiregulin in the development of colorectal cancer

**DOI:** 10.1002/cam4.416

**Published:** 2015-01-30

**Authors:** Sung Ouk Nam, Fusanori Yotsumoto, Kohei Miyata, Satoshi Fukagawa, Hiromi Yamada, Masahide Kuroki, Shingo Miyamoto

**Affiliations:** 1 Department of Obstetrics and Gynecology, Faculty of Medicine, Fukuoka UniversityFukuoka, Japan; 2 Central Research Institute for Advanced Molecular Medicine, Fukuoka UniversityFukuoka, Japan; 3 Department of Biochemistry, Faculty of Medicine, Fukuoka UniversityFukuoka, Japan

**Keywords:** Aerobic glycolysis, amphiregulin, colorectal cancer, glucose metabolism, MLX

## Abstract

Colorectal cancer (CRC) is one of the most frequently occurring cancers with high morbidity and mortality worldwide. Amphiregulin (AREG), a member of the epidermal growth factor family and a rational target for CRC therapy, is essential for the three-dimensional structure of tumor formation. To clone the genes associated with increased AREG expression, we performed a cDNA microarray analysis in two CRC cell lines undergoing two-dimensional (2DC) and three-dimensional culture (3DC). Upregulated (>2.0-fold) and downregulated (<0.5-fold) genes in 3DC compared with 2DC were selected. Pathway analysis using DAVID based on the Kyoto Encyclopedia of Genes and Genomes (KEGG) pathway databases revealed a number of genes involved in glycolysis. In CRC cells, glucose elevated the expression of GLUT1 and AREG as well as the activity of the hypoxia-inducible factor 1 (HIF-1) luciferase reporter promoter. The suppression of AREG expression reduced the uptake of glucose and production of lactate. Luciferase assay identified a critical regulatory region for AREG expression between −130 and −180 bp upstream of the start site, which contained a carbohydrate response element (ChoRE). Max-like protein X (MLX) bound to ChoRE and enhanced the expression of AREG. Together these data suggest that AREG plays a pivotal role in the development of CRC through activation of the Warburg effect.

## Introduction

Colorectal cancer (CRC) shows high mortality and morbidity worldwide 1. Various molecular targeted agents such as anti-vascular endothelial growth factor (VEGF) monoclonal antibody (bevacizumab) and anti-epidermal growth factor receptor (EGFR) monoclonal antibody (cetuximab) have been developed for CRC therapy 2,3. Although treatment with bevacizumab provides a favorable outcome, it is associated with fatal adverse side effects including hypertension and perforation of the digestive tract 3. Furthermore, cetuximab has a reduced clinical effect in CRC patients with *KRAS* mutations 2. Although the prognosis of CRC has been improved by the development of operative procedures and therapeutic agents, the mortality rate of CRC patients is still over 600,000 and the morbidity rate in CRC patients is also increasing in gastrointestinal cancer 4,5. Consequently, the development of novel targeted agents for CRC therapy is required.

Many cancer cells exhibit elevated uptake of glucose and production of lactate under hypoxic conditions, which is known as aerobic glycolysis or the Warburg effect, resulting in enhanced tumor cell growth 6–8. The Warburg effect is associated with the upregulated expression of many molecules, including GLUT family members, hexokinases (HKs), pyruvate dehydrogenase kinases (PDKs), and lactate dehydrogenases (LDHs) 8. Emerging evidence has revealed that oncogenes and tumor suppressors, such as PTEN, PI3K/Akt/mTOR, HIF-1*α*, AMPK, p53, EGFR, ERK1/2, PMK2, RAS, and Myc, regulate altered energy metabolism in cancer 9–11. A previous report demonstrated that the expression levels of GLUT1, HKs, and PDKs were significantly elevated in CRC 12. In addition to these molecules, several oncogenes and tumor suppressors involved in the Warburg effect were attributed to the development of CRC 13. However, the mechanistic details underlying the causes and subsequent processes of the Warburg effect in CRC have remained unclear.

Amphiregulin (AREG) is a member of the EGF family that contributes to cancer proliferation and progression 14. AREG is secreted through ectodomain shedding mainly via the actions of a disintegrin and metalloproteinase (ADAM) family 15. Cleaved AREG binds to and transactivates its receptor, EGFR 16, and functions as a growth factor for many cell types including keratinocytes, mammary epithelial cells, hepatocytes, and intestinal epithelial cells 17–19. In addition, AREG participates in wound healing of damaged colonic mucosa 20, and AREG expression in CRC is significantly associated with an increased frequency of local lymph node involvement 21. In vitro analyses have validated AREG as a rational target for CRC therapy 22. Accordingly, AREG may be a key molecule involved in the acquisition of a malignant phenotype in CRC.

To investigate the significance of AREG in the development of CRC and to elucidate the interaction between AREG and glucose metabolism in CRC, we investigated genes involved in the enhanced expression of AREG. Pathway analysis indicated the identified genes were implicated in glycolysis. Our results demonstrated that glucose induced the expression of AREG through transcriptional regulation by Max-like protein X (MLX), leading to the development of CRC.

## Materials and Methods

### Cell lines and culture

The HCT116, HT29, LoVo, WiDr, CoLo201, and LS180 cell lines were obtained from the American Type Culture Collection (Manassas, VA). All cells were maintained in RPMI1640 medium supplemented with 10% fetal bovine serum (FBS) (ICN Biomedicals, Irvine, CA), 100 U/mL of penicillin G, and 100 *μ*g/mL of streptomycin (Invitrogen Corp., Carlsbad, CA) in a humidified atmosphere of 5% CO_2_ at 37°C.

### Three-dimensional culture

Cultured cells were detached with trypsin-EDTA, washed three times with serum-free medium, and suspended at a final concentration of 2 × 10^5^ cells/1.5 mL. Aliquots (1.5 mL) were applied to the wells of six-well plates precoated with 1.5 mL/well Matrigel (Becton Dickinson, Franklin Lakes, NJ). Cells were then cultured in RPMI1640 medium containing 10% FBS under each experimental condition. Cells were retrieved from colonies using a BD Cell Recovery Solution (Becton Dickinson).

### Real-time quantitative PCR

RNA extraction was performed using Trizol (Invitrogen) and first-strand cDNAs were synthesized from equal amounts of total RNA (1 *μ*g/reaction) with a PrimeScript II first-strand cDNA synthesis kit (Takara Bio, Otsu, Shiga, Japan) in a total volume of 20 *μ*L, as described by the manufacturer's protocol. Synthesized cDNAs were used for real-time PCR. Real-time PCR was performed using the Applied Biosystems 7500 Real-Time PCR Systems (Applied Biosystems, Foster City, CA). The TaqMan quantitative PCR was carried out using primer pairs, and TaqMan probes for each EGFR ligand and glyceraldehyde-3-phosphate dehydrogenase (GAPDH) were as follows: AREG: Hs00950669_m1; heparin-binding epidermal growth factor-like growth factor (HB-EGF): Hs00181813_m1; transforming growth factor alpha (TGF-*α*): Hs00608187; epidermal growth factor (EGF): Hs01099999_m1; GAPDH: Hs02758991_g1; and solute carrier family 2 (facilitated glucose transporter), member 1 (GLUT1): Hs00892681_m1. Serial 1:10 dilutions of plasmid DNA containing each target cDNA (10^7^–10^1^ copies/*μ*L) were analyzed and served as standard curves, from which we determined the rate of changes of the threshold cycle values. Copy numbers of the target cDNAs were estimated from the standard curves. The expression of HIF-1*α*, LDH-A, LDH-B, HKI, HKII, PDK2, and PDK4 gene transcripts was determined using SYBR Green PCR Master Mix (Applied Biosystems). Each PCR was carried out according to the manufacturer's instructions. Forward and reverse primers were designed with Primer Blast online as shown in Table S1. To evaluate mRNA levels, we used the mRNA expression index, which reflects the relative mRNA expression level standardized by GAPDH. The mRNA expression index was calculated as follows (in arbitrary units): mRNA expression index = (copy number of each mRNA/copy number of GAPDH mRNA) × 10,000 arbitrary units.

### Soluble AREG, HB-EGF, EGF, and TGF-*α* in cell culture media

Cells were incubated for 48 h, and the levels of EGFR ligands in culture medium were determined using a commercially available sandwich ELISA (R&D Systems Inv., Minneapolis, MN) according to the manufacturer's instructions. All samples were normalized by cell numbers. Each mean value was considered representative of corresponding culture media.

### Plasmid construction and reporter gene assay

The *AREG* promoter fragment was prepared from genomic DNA from HCT116 cells. A region 840 bp upstream and 210 bp downstream of the AREG transcription start site was amplified using PCR, and subcloned into pGL4.12 (Promega, Madison, WI) vector. For the promoter assays, the *AREG* promoter was amplified by PCR and digested with *Nhe*I and *Hin*dIII restriction enzymes (Toyobo, Osaka, Japan). Each *AREG* promoter fragment was prepared by subcloning of the promoter fragments −840/+210, −680/+210, −380/+210, −180/+210, and −40/+210 into the *Nhe*I/*Hin*dIII sites of pGL4.12 (*luc2CP*) vector (Promega). The ChoRE deletion mutant vector was prepared by Inverse PCR. All primer sequences used for subcloning are presented in Table S2.

For luciferase reporter assays, HCT116 cells were grown in 12-well plates and transfected with *AREG* promoter-driven luciferase reporter plasmids (2 *μ*g/well) and the SV40-driven pRL control plasmid (0.005 *μ*g/well) using FuGENE® HD Transfection Reagent (Promega). Cells were harvested 48 h after transfection, and firefly and Renilla luciferase activity were determined using the Dual Luciferase Assay kit (Promega) and TriStar LB 941 Luminometer (Berthold Technologies, Bad Wildbad, Germany). Transcription factor-binding sites were predicted by MatInspector (http://www.genomatix.de). The Cignal Lenti-HIF Reporter system (Qiagen, Venlo, The Netherlands) was used to stably transduce HCT116 cells with HIF-regulated firefly luciferase constructs using SureENTRY Transduction Reagent (Qiagen). HCT116 cells were grown to 60–70% confluence and infected for 24 h according to the manufacturer's protocol using 8 *μ*g/mL SureENTRY Transduction Reagent (Qiagen). Cells were selected by puromycin and positive clones were expanded. For analysis of HIF activity, cells at 70% confluence were lysed and dual luciferase activity was analyzed.

### siRNA transfection and anticancer drug

Cells (5 × 10^5^) were seeded on 6-cm plates (50–60% confluence). Control siRNA (Stealth RNAi Negative Control) or siRNAs for AREG, EGFR, and GLUT1 (Invitrogen, Carlsbad, CA) or MLX (Sigma) were transfected into cells using Lipofectamine RNAi-MAX Transfection Reagent (Invitrogen) according to the manufacturer's instructions. The final concentration of each siRNA for transfection was 50 nmol/L. Erlotinib, an EGFR tyrosine kinase inhibitor, was kindly provided by F. Hoffmann–La Roche (Basel, Switzerland). Cells (5 × 10^5^) were seeded in 6-cm plates (50–60% confluence) in the presence of 20 *μ*mol/L of Erlotinib. After incubation for 24 h, cells were detached from plates with trypsin-EDTA and replated into six-well plates precoated with 1.5 mL/well of Matrigel (Biocoat Cellware; Becton Dickinson) for three-dimensional culture (3DC).

### Measurement of glucose and lactate levels

Cells were seeded onto six-well plates precoated with 1.5 mL/well of Matrigel (Biocoat Cellware; Becton Dickinson) using glucose-free RPMI1640 medium. Glucose-free RPMI1640 medium was replaced with RPMI1640 medium containing glucose 12 h after cells were seeded and the cells were incubated for another 24 h. After incubation, conditioned medium was collected and cells were retrieved from colonies using a BD Cell Recovery Solution (Biocoat Cellware; Becton Dickinson) and lysed with 0.2 mL Passive Lysis Buffer (Promega). Glucose content was measured using a Glucose Assay Kit II (BioVision, CA) according to the manufacturer's protocol. Lactate was measured with a Lactate Assay Kit II (Bio Vision) according to the manufacturer's protocol. The glucose and lactate values were normalized to the protein concentration determined using a Bradford assay kit (Bio-Rad, Tokyo, Japan).

### Expression array analysis

Total RNA was extracted from HCT116 and HT29 cells cultured under 2DC or 3DC using Trizol. Gene expression arrays were conducted using a Whole Human Genome DNA microarray (4×44K) v2 (Agilent Technologies, Santa Clara, CA) and analyzed by Feature Extraction software (Agilent Technologies).

### Chromatin immunoprecipitation assay

The cDNA encoding full-length MLX was cloned from HCT116 cells using PCR. Amplicons were then subcloned into a pcDNA3.1/V5/His TOPO vector (Invitrogen) under control of the CMV promoter. HCT116 cells were grown in 10-cm plates and transfected with pcDNA3.1/V5/His TOPO vector-MLX using FuGENE® HD Transfection Reagent (Promega). HCT116-MLX cells were seeded onto 15-cm plates and medium was replaced with RPMI1640 medium containing 5.5 or 25 mmol/L glucose after cells became subconfluent. After 12 h incubation, cells were crosslinked by adding 1% formaldehyde in phosphate buffered saline (PBS) for 15 min at room temperature. Glycine was added at a final concentration of 0.125 mol/L for 5 min at room temperature to terminate the cross-linking reaction. Cells were harvested using a cell scraper after adding 2 mL cold PBS containing 1× Protease Inhibitor Cocktail II. Nuclear extraction, sonication, immunoprecipitation, crosslink reversal, and DNA cleanup were performed using Millipore EZ-Magna ChIPTM A (Millipore, Billerica, MA) according to the manufacturer's protocol. Immunoprecipitation was performed with anti-His monoclonal antibody (Abcam, Cambridge, UK), mouse IgG, anti-tri-methyl-histone H3 (Lys 4) rabbit antibody or normal rabbit IgG (CST Japan, Tokyo, Japan). DNA fragments were quantified by real-time PCR using SYBR Premix Ex Taq (Takara) with the primers listed in Table S2. DNA eluted from the DNA–protein complex before immunoprecipitation was used as “input.” The relative value of DNA fragments was calculated by extrapolation from a standard curve of input DNA dilutions.

### Statistical analysis

The statistical significance of differences between values was assessed using the Mann–Whitney *U* test. A value of *P* < 0.05 was considered statistically significant.

## Results

### Identification of genes associated with enhanced AREG expression in CRC

**Figure 1:**
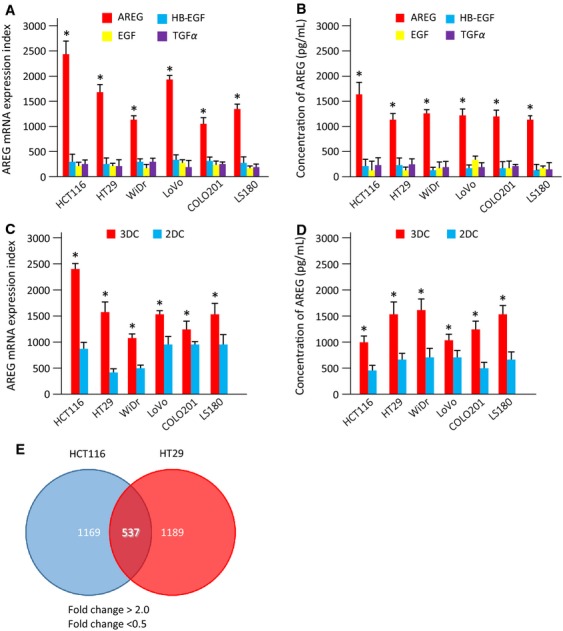
Screening of genes involved in CRC tumorigenesis. (A) mRNA expression indexes of EGFR ligands in CRC cell lines under three-dimensional culture (3DC). The mRNA expression levels of AREG (red bar), HB-EGF (blue bar), EGF (yellow bar), and TGF*α* (purple bar) are shown. Data were measured in triplicate and represent mean ± SD. *P < 0.05 versus other EGFR ligands. (B) Levels of soluble EGFR ligands in culture medium under 3DC. Levels of AREG, HB-EGF, EGF, and TGF*α* proteins per cell were measured by ELISA. Data were measured in triplicate and represent mean ± SD. *P < 0.05 versus other EGFR ligands. (C) Expression of AREG mRNA in CRC cell lines under 3DC or two-dimensional culture (2DC). The mRNA expression levels of AREG were coded as follows: 3DC, red bar; 2DC, blue bar. Data were measured in triplicate and represent mean ± SD. *P < 0.05 versus 2DC. (D) Levels of soluble AREG protein under 2DC or 3DC conditions. The expression levels of AREG protein per cell were measured by ELISA. *P < 0.05 versus 2DC. Data were measured in triplicate and represent mean ± SD. (E) Upregulated (>twofold) and downregulated (<0.5-fold) genes detected by expression microarray analysis. Venn diagrams show the number of common genes in HCT116 and HT29 cells with altered expression in 3DC compared with 2DC. Of these, 537 genes were shared between the two CRC cell lines. The microarray data were obtained from the Gene Expression Omnibus (GEO) database (GEO accession numbers: GSE56738). CRC, colorectal cancer; EGFR, epidermal growth factor receptor; AREG, amphiregulin; HB-EGF, heparin-binding epidermal growth factor-like growth factor; TGF*α*, transforming growth factor alpha; ELISA, enzyme-linked immunosorbent assay.

To address the significance of AREG as a target for CRC therapy, we examined the mRNA expression and supernatant protein levels of EGFR ligands from CRC cell lines (HCT116, HT29, LoVo, WiDr, CoLo201, and LS180) under 3DC conditions. Results showed a significant increase in AREG mRNA expression and prominent secretion of AREG in 3DC media compared with other EGFR ligands examined (Fig.1A and B). Next, to evaluate alterations in AREG expression associated with tumorigenesis, we examined AREG mRNA expression and supernatant protein levels of CRC cell lines in 2DC and 3DC. AREG mRNA levels and secreted AREG protein levels were significantly increased in 3DC compared with 2DC (Fig.1C and D). These results suggested that AREG might play an important role in CRC tumorigenesis compared with other EGFR ligands.

To identify specific pathways and genes associated with CRC tumorigenesis, we examined gene expression changes in HCT116 and HT29 cells when cultured in 3DC compared with 2DC. We identified 1169 and 1189 upregulated (>twofold; *P* < 0.05) and downregulated (<0.05-fold; *P* < 0.05) genes in HCT116 and HT29 cells, respectively, by microarray analysis. The microarray data can be found in the Gene Expression Omnibus (GEO) database (GEO accession numbers: GSE56738). Among all identified genes, 537 genes were commonly regulated in both HCT116 and HT29 cell lines (Fig.1E). To examine how the expressed genes and their specific pathways were associated with enhanced AREG expression in CRC tumorigenesis, we performed pathway analysis using DAVID based on Kyoto Encyclopedia of Genes and Genomes (KEGG) and BIOCARTA pathway databases. KEGG, as well as BIOCARTA, pathway analysis indicated that the commonly regulated genes were involved in two specific pathways: glycolysis and oocyte maturation (Table1). This study focused on the glycolysis pathway, because aerobic glycolysis is important for tumor cell growth. Together, these findings indicated that the glucose metabolism pathway was associated with enhanced AREG expression in CRC, and suggested that genes related to glucose metabolism play an important role in AREG function in CRC tumorigenesis.

**Table 1:** Functional pathways of upregulated (>twofold) or downregulated (<0.5-fold) genes in HCT116 and HT29 cells cultured in 3DC compared with 2DC

Term	Count	*P*-value
KEGG pathway database		
Cell cycle	19	1.2 E−8
Oocyte meiosis	14	1.4 E−5
Glycolysis/gluconeogenesis	8	1.6 E−3
Progesterone-mediated oocyte maturation	9	3.2 E−3
Systemic lupus erythematous	9	7.5 E−2
Antigen processing and presentation	4	9.1 E−2
BIOCARTA		
How progesterone initiates the oocyte maturation	4	9.9 E−1
Glycolysis pathway	3	9.8 E−1
Role of Ran in mitotic spindle regulation	9	9.5 E−1
Stathmin and breast cancer resistance to antimicrotubule agents	14	9.3 E−1

3DC, three-dimensional culture; 2DC, two-dimensional culture; KEGG, Kyoto Encyclopedia of Genes and Genomes.

### AREG regulation of the Warburg effect via HIF-1

**Figure 2:**
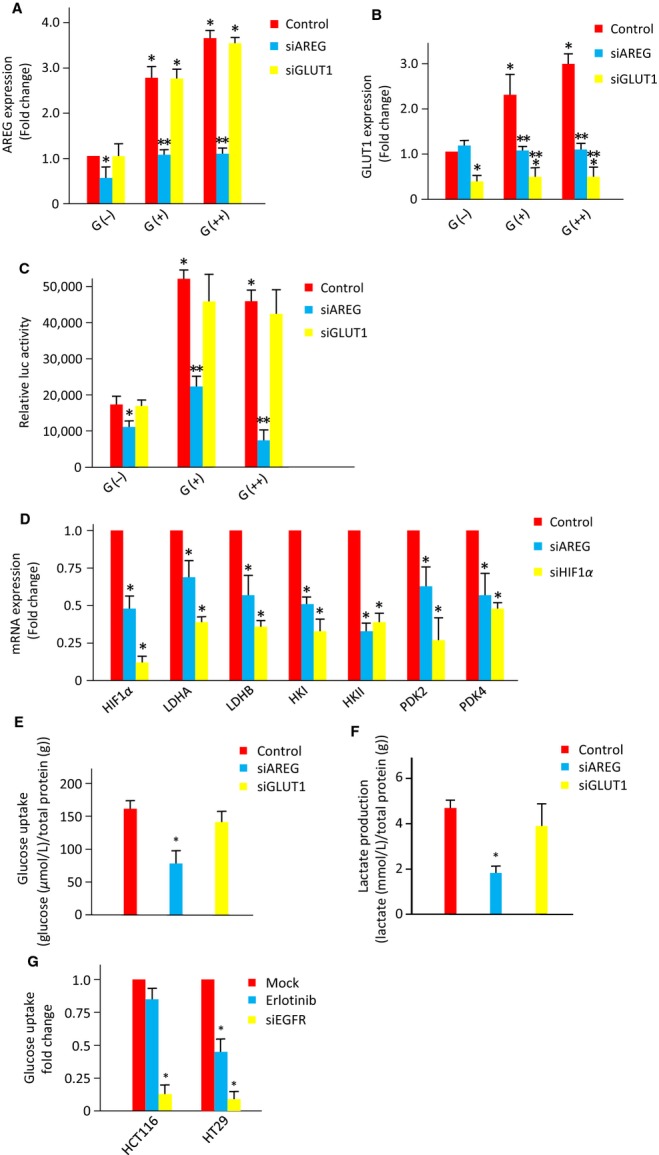
Function of AREG and GLUT1 in glucose metabolism in CRC. AREG (A) and GLUT1 (B) mRNA expression in HCT116 cells transfected with AREG, GLUT1, or control siRNA and cultured in glucose-free (G(−)), 5.5 mmol/L glucose (G(+)), or 25 mmol/L glucose (G(++)) medium. Data are expressed as fold change relative to G(−) medium. Data were measured in triplicate and represent mean ± SD. *P < 0.05 versus the control G(−). **P < 0.05 versus each control. (C) Dual luciferase reporter assays in lysates from HCT116 cells transfected with the Cignal Lenti-HIF Reporter system. After Lenti-HIF was introduced into HCT116 cells, AREG, or GLUT1 siRNA was transfected. Data were measured in triplicate and represent mean ± SD. *P < 0.05 versus the control G(−). **P < 0.05 versus each control. (D) mRNA expression indexes of genes (HIF-1*α*, LDH-A, LDH-B, HKI, HKII, PDK2, and PDK4) which are directly involved in the Warburg effect in HCT116 cells under three-dimensional culture. The mRNA expression levels of control (red bar), siAREG (blue bar), and siHIF-1*α* (yellow bar) are shown. Experiments were done in triplicates and represent the mean ± SD. *P < 0.05 versus control. The concentration of glucose (E) or lactate (F) was measured in HCT116 cells transfected with AREG, GLUT1, or control siRNA. The concentration of glucose (G) was measured in HCT116 cells transfected with EGFR or control siRNA and Erlotinib. Data were measured in triplicate and represent mean ± SD. *P < 0.05 versus controls. AREG, amphiregulin; GLUT1, glucose transporter member 1; HIF, hypoxia-inducible factor; HK, pyruvate dehydrogenase kinases; LDH, lactate dehydrogenase; siRNA, small interfering RNA.

To validate the microarray results, we analyzed the expression of various genes involved in the Warburg effect using HCT116 and HT29 cells cultured in 3DC and 2DC by quantitative real-time-PCR. The expression of HIF-1*α*, HK2, LDH-A, PDK2, PDK4, GLUT1, and SGLT1 were significantly increased in 3DC compared with 2DC, while no significant differences in the expression of PDK3, GLUT2, GLUT3, GLUT4, and SGLT2 were observed between 2DC and 3DC in both HCT116 and HT29 cells. Expression of LDH-B was increased in HCT116 cells and HK1 and PDK1 was increased in HT29 cells in 3DC when compared with 2DC (Figs. S1 and S2). To investigate the association between AREG expression and glucose metabolism, we examined the expression of AREG and GLUT1 in HCT116 cells cultured under various glucose concentrations. Increased concentrations of glucose enhanced the expression of GLUT1 and AREG in HCT116 cells (Fig.2A and B) and stimulated luciferase activity driven by the HIF-1 promoter (Fig.2C). Transfection of AREG siRNA in HCT116 cells reduced the glucose-mediated enhanced expression of AREG and GLUT1 (Fig.2A and B) and diminished glucose-mediated stimulation of luciferase activity of the HIF-1 reporter (Fig.2C). Transfection with GLUT1 siRNA suppressed the expression of GLUT1, although no significant change in glucose-mediated AREG expression or HIF-1 luciferase activity was detected (Fig.2A–C). The transfection of siRNA for AREG or GLUT1 decreased the protein expression levels of AREG and GLUT1, respectively (Fig. S3). We examined the mRNA expression of HIF-1*α*, LDH-A, LDH-B, HKI, HKII, PDK2, and PDK4 in 2DC and 3DC after the knock down of AREG or HIF-1*α* using siRNA. In 2DC or 3DC, the expression of HIF-1*α*, LDH-A, LDH-B, HKI, HKII, PDK2, and PDK4 were suppressed upon transfection with siRNA for AREG or HIF-1*α*. These results suggest that the expression levels of genes directly involved in the Warburg effect are regulated by AREG in colon cancer. The introduction of siRNA for AREG into HCT116 cells inhibited the uptake of glucose and the production of lactate. However, the inhibition of GLUT1 did not significantly inhibit the uptake of glucose and the production of lactate but induced a tendency to suppress the uptake of glucose. These results suggest that the suppression of GLUT1 expression is not enough to suppress the uptake of glucose or that there is a positive transport of glucose mediated by AREG (Fig.2E and F). Additionally, we showed that suppression of EGFR expression using an EGFR inhibitor and siRNA suppressed the uptake of glucose (Fig.2G). These results suggested that glucose promoted the expression of AREG, which subsequently activated HIF-1 and induces the expression of GLUT1. Furthermore, these result suggested that the AREG-EGFR pathway is partially involved in the expression of GLUT1 and the Warburg effect. Thus, AREG may regulate the Warburg effect in CRC.

### Identification of the transcriptional regulatory region of AREG

**Figure 3:**
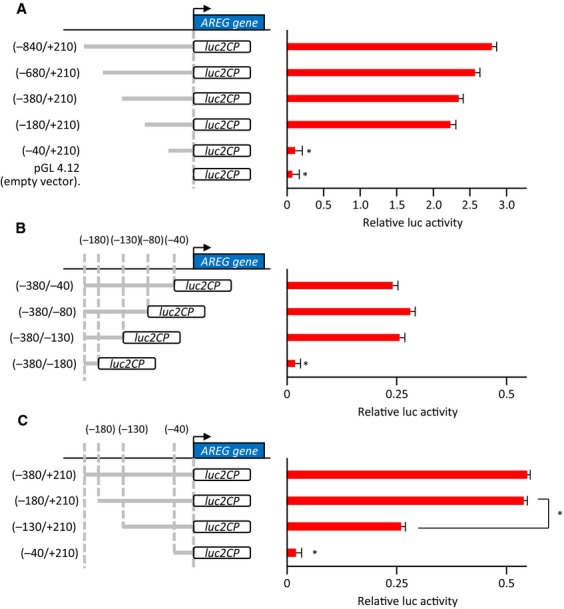
The transcriptional regulatory element of amphiregulin (AREG). (A) Dual luciferase reporter assays in HCT116 cells transfected with AREG promoter constructs. Activation of the reporter gene was calculated by the ratio of firefly luciferase activity to Renilla luciferase activity. Data were measured in triplicate and represent mean ± SD. *P < 0.05 versus the −180/+210, −380/+210, −680/+210, or −840/+210 constructs. (B) Dual luciferase reporter assays using a second set of AREG promoter constructs. Data were measured in triplicate and represent mean ± SD. *P < 0.05 versus the −380/−40, −380/−80, or −380/−130 constructs. (C) Dual luciferase reporter assays using a third set of AREG promoter constructs. Data were measured in triplicate and represent mean ± SD. *P < 0.05 versus the −380/+210 or −180/+210 construct.

To identify the transcriptional factors that directly regulate AREG expression, we examined the transcriptional region controlling AREG expression using a luciferase reporter assay. Approximately 1.05 kbp was cloned from the transcriptional start site (TSS) of the AREG gene, which is conserved among mammalian species, and luciferase reporter vectors containing various fragments of the cloned region were examined. Luciferase assays showed that reporter vectors containing the promoter fragments −180/+210 bp from the AREG TSS (pGL/AREG_−180/+210_) (Fig.3A) and −380/−130 bp from the AREG TSS (pGL/AREG_−380/−130_) (Fig.3B) exhibited a 20-fold increase in luciferase activity compared with that of pGL/AREG_−40/+210_ or pGL/AREG_−380/−180_, respectively.

To confirm that the transcriptional regulatory region of AREG was located between −180 and −130 bp from the TSS, we generated another set of luciferase constructs. Luciferase activity of pGL/AREG_−130/+210_ was significantly suppressed compared to pGL/AREG_−180/+210_ (Fig.3C), indicating that the sequence from −180 to −130 bp from the TSS of AREG is a regulatory region critical for AREG expression.

### MLX transcription factor binds the transcriptional regulatory element of AREG

**Figure 4:**
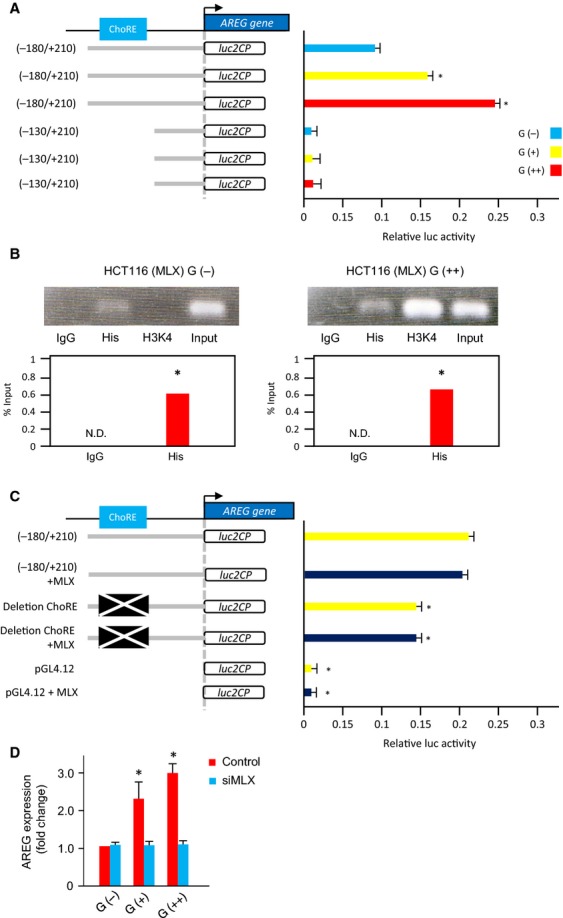
Transcriptional regulation of AREG expression by the MLX transcriptional factor. (A) Dual luciferase reporter assays in HCT116 cells transfected with −180/+210 or −130/+210 AREG promoter constructs and cultured in glucose-free (G(−)), 5.5 mmol/L glucose (G(+)), or 25 mmol/L glucose (G(++)) RPMI1640 medium. Data were measured in triplicate and represent mean ± SD. *P < 0.05 versus G(−). (B) ChiP analysis of MLX binding to the AREG promoter. HCT116 cells were transfected with the pcDNA3.1/V5/His TOPO vector containing full-length MLX (HCT116-MLX) and cultured under glucose conditions as indicated. DNA–protein complexes were immunoprecipitated by His or IgG antibody and PCR was performed using primers to amplify the AREG promoter. Input samples were used as a control. *P < 0.05 versus the IgG control. N.D., not determined. (C) Luciferase assays using the ChoRE deletion construct. Dual luciferase reporter assays in HCT116 cells transfected with the wild-type −180/+210 or pGL4.12-ΔChoRE (deletion mutant of ChoRE) construct and MLX as indicated. Data represent the mean ± SD measured in triplicate. *P < 0.05 versus −180/+210 with or without transfection of MLX gene. (D) AREG mRNA expression in HCT116 cells transfected with MLX siRNA and cultured in G(−), G(+), and G(++) medium. Data are expressed as fold change relative to G(−) medium. Data were measured in triplicate and represent mean ± SD. *P < 0.05 versus the control G(−). AREG, amphiregulin; ChoRE, carbohydrate response element; PCR, polymerase chain reaction; siRNA, small interfering RNA; MLX, Max-like protein X.

In silico analysis indicated that 10 transcription factors putatively bound transcriptional sites between −180 and −130 bp in the AREG gene (Fig. S4). Among these transcription factors, we focused on MLX, which binds to carbohydrate response elements (ChoREs) involved in glucose metabolism. We performed luciferase assays using a reporter vector containing a CHoRE and found that increased glucose concentrations markedly enhanced luciferase activity of the CHoRE-containing constructs (Fig.4A). To assess the ability of MLX to bind the promoter region of AREG, we performed chromatin immunoprecipitation (ChIP) analysis in HCT116 cells transfected with cDNA encoding the full-length MLX gene (HCT116-MLX). qRT-PCR analysis revealed a significant increase in MLX binding to the AREG promoter in response to increased glucose levels, compared with control IgG (Fig.4B). In addition, there was increased transcriptional activation of the AREG gene in the presence of high levels of glucose, as measured by histone H3 lysine 4 methylation (H3K4) (Fig.4B).

To further elucidate the role of ChoRE in the expression of AREG, we examined the activity of a luciferase reporter driven by the *AREG* promoter region with the ChoRE deleted (pGL/AREG_−180/+210_/D-ChoRE), and found a significant decrease in luciferase activity of the mutant construct compared with the wild-type *AREG* promoter sequence (pGL/AREG_−180/+210_) (Fig.4C). The presence or absence of cotransfected full-length MLX had no significant effect on the luciferase activity of three different reporters (pGL/AREG_−180/+210_, pGL/AREG_−180/+210_/D-ChoRE and pGL4.12) (Fig.4C). Finally, transfection of MLX siRNA suppressed the enhanced AREG expression mediated by increased glucose levels (Fig.4D). Together these data indicate that ChoRE is one of the transcriptional regulatory elements of AREG and that glucose causes an increase in AREG expression through the direct binding of MLX to ChoRE in the AREG promoter.

## Discussion

**Figure 5:**
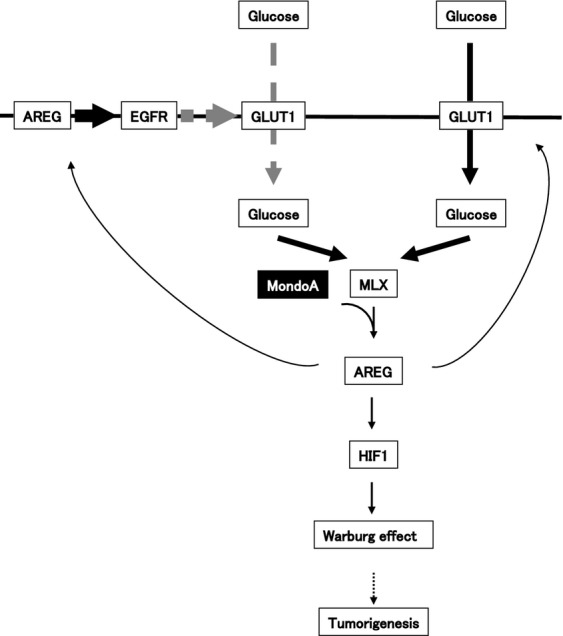
Schematic model of AREG in the Warburg effect and tumorigenesis. The influx of glucose is mainly mediated by GLUT1, a transporter molecule. The expression of GLUT1 was shown to be partly controlled by the AREG-EGFR pathway. The MLX transcription factor, which possibly forms a complex with MondoA, directly binds to the promoter region of AREG via ChoRE, resulting in enhanced AREG expression. AREG may transactivate EGFR and subsequently activate HIF-1, resulting in the induction of many genes associated with the Warburg effect. The MLX-MondoA complex likely promotes the expression of many genes associated with the Warburg effect, independent of HIF-1. The Warburg effect regulated by AREG may be involved in CRC tumorigenesis. AREG, amphiregulin; GLUT1, glucose transporter member 1; HIF, hypoxia-inducible factor; ChoRE, carbohydrate response element; CRC, colorectal cancer; EGFR, epidermal growth factor receptor; MLX, Max-like protein X.

A schematic depicting the proposed model of a role for AREG in the Warburg effect and tumorigenesis based on the results from this study is shown in Figure5. The uptake of glucose in CRC occurs via GLUT1. Following the influx of glucose into cells, MLX, which forms a complex with MondoA, directly binds to the promoter of the AREG gene and upregulates AREG expression in CRC. In addition, the increase in AREG expression possibly enhances the activity of HIF-1 through the phosphorylation of EGFR. The uptake of glucose is carried out by both the GLUT and SGLT families. However, in this experiment, only GLUT1 expression was elevated compared with other GLUTs in colon cancer cell lines. In addition, both the expression of GLUT1 and the uptake of glucose was decreased by the suppression of AREG expression. However, the suppression of GLUT1 did not significantly affect the uptake of glucose and AREG expression. Therefore, we think that GLUT1 may have another function besides that of a passive transporter. We showed that suppression of EGFR expression using an EGFR inhibitor and siRNA suppressed the uptake of glucose. From these results, we could show that the expression of GLUT1 is partly controlled by the AREG-EGFR pathway. Furthermore, these results suggest that the AREG-EGFR pathway is partially involved in the expression of GLUT1 and the Warburg effect. Finally, HIF-1 induces the production of lactate via the expression of HKs, LDHs, PDKs and GLUT family members. Thus, AREG regulates the Warburg effect in the development of CRC.

Glucose is rapidly converted into glucose-6-phosphate in cells, which in turn activates basic helix-loop-helix-leucine zipper transcriptional factors ChREBP and MondoA that heterodimerize with MLX. ChREBP-MLX and MondoA-MLX mediate a majority of the glucose-induced transcriptional responses by binding to target gene promoters that contain ChoREs 23. MondoA-MLX and ChREBP-MLX are key regulators of genes involved in energy metabolism, especially glycolysis or lipogenesis 24–27. The expression of HKII, LDH-A, or 6-phosphofructo-2-kinase/fructose-2, 6-bisphosphatase (PFKFB3) is activated by MondoA-MLX. MondoA-MLX also binds to ChoREs in the promoter of the thioredoxin-interacting protein (Txnip) and induces the expression of Txnip, which is an important regulator of glucose metabolism that functions by inhibiting cellular uptake of glucose 28,29. In hypoxic conditions, HIF-1 promotes the expression of HKs, PDKs, LDHs, and GLUT family members 30. In this study, the influx of glucose augmented the expression of AREG and an increase in AREG expression mediated by MLX that subsequently activated HIF-1 in CRC. According to these data, it is plausible that the influx of glucose induces the expression of HKs, PDKs, and LDHs involved in the Warburg effect through direct and indirect transcription mediated by MLX and enhance CRC growth.

Recently, type 2 diabetes was linked to the increased risk of developing colorectal, pancreatic liver, kidney, endometrial, and breast cancer 31–33. GLUT1, AMPK, HIF-1, and PI3K/Akt/mTOR are associated with type 2 diabetes pathogenesis and might be rational targets for diabetes therapy 34–36. These molecules are also associated with the Warburg effect in CRC. Increasing evidence reveals that altered energy metabolism has a similar consequence to carcinomas at the cellular and molecular level 37. MLX plays a key role in glucose metabolism in pancreatic and liver tissues 38,39, and the overexpression of GLUT1 is involved in the pathogenesis of colon, pancreatic, and liver cancer 40. In addition, AREG is recognized as a therapeutic target for colon, pancreatic, liver cancer, and renal cell carcinoma 22,41. Metformin, an oral antidiabetic drug that suppresses insulin resistance, functions as a growth inhibitor of epithelial cells by reducing mTOR activity 42–44. In a meta-analysis, the use of metformin was associated with a significantly decreased rate of CRC and pancreatic cancer recurrence 45. In addition to the development of CRC, therefore, the Warburg effect regulated by AREG might be involved in the development of CRC as well as the development of pancreatic and liver cancer.

The persistent activation of aerobic glycolysis might lead to cancer progression. Therefore, the inhibition of cellular glycolytic capacity appears to be attributable to an anticancer effect on malignant cells. Inhibitors against genes involved in the Warburg effect have been developed as anticancer agents 46,47. For example, inhibitors of GLUT1, HK, LDH, PDK, and MCT have been used to treat patients with cancer in preclinical and clinical studies. To date, clinical studies have been impaired by significant pancreatic and hepatic toxicities 48. However, several reports indicate that combinational treatment with conventional anticancer agents has a synergistic inhibitory effect on tumor growth 49,50. An increased understanding of cancer metabolic profiles provides hope that a novel class of therapeutic agents may be developed for cancer therapy.

The current study demonstrated that AREG is involved in tumorigenesis and glucose metabolism and therefore is validated as a promising target for CRC therapy. Therefore, in the near future, the development of an inhibitor for AREG would improve the outcome in patients with CRC, when used as a combinational treatment with conventional anticancer agents or antidiabetic drugs, such as metformin.

## Supporting Information

Data S1 Supplementary Materials and Methods. **Figure S1.** Alterations in the expression of genes involved in the Warburg effect in 2DC (blue bars) compared with 3DC (red bars) in HCT116 cells. (A) HIF-1*α*, (B) HK1, (C) HK2, (D) LDH-A, (E) LDH-B, (F) PDK1, (G) PDK2, (H) PDK3, (I) PDK4, (J) GLUT1, (K) GLUT2, (L) GLUT3, (M) GLUT4, (N) SGLT1, and (O) SGLT2. Data were measured in triplicate and represent mean ± SD. **P* < 0.05 versus 2DC. **Figure S2.** Alterations in the expression of genes involved in the Warburg effect in 2DC (blue bars) and 3DC (red bars) in HT29 cells. (A) HIF-1*α*, (B) HK1, (C) HK2, (D) LDH-A, (E) LDH-B, (F) PDK1, (G) PDK2, (H) PDK3, (I) PDK4, (J) GLUT1, (K) GLUT2, (L) GLUT3, (M) GLUT4, (N) SGLT1, and (O) SGLT2. Data were measured in triplicate and represent mean ± SD. **P* < 0.05 versus 2DC. **Figure S3.** Protein levels of AREG or GLUT1 under 3DC for 48 h after transfection with gene specific siRNAs into HCT116 cells. (A) The levels of AREG proteins in culture media of HCT116 cells were measured by ELISA. **P* < 0.05. (B) Total GLUT1 proteins in HCT116 cells were detected by immunoblotting. GAPDH was used as internal control. Representative data of three independent experiments are shown. **Figure S4.** The primary structure of the −180/−130 promoter region of AREG. In silico analysis reveals the putative molecules bound to the promoter regions and their sequences (highlighted in red). **Table S1.** Forward and reverse primers of genes for real-time PCR amplification used in this study. **Table S2.** Primers used in this study.
