# Supplementary material for: Warburg effect regulated by amphiregulin in the development of colorectal cancer
Source: Cancer Med. 2015 Jan 30;4(4):575–87. doi: 10.1002/cam4.416 (PMC4402072; doi:10.1002/cam4.416)
Supplement: Supplementary file 1 [file cam40004-0575-sd1.ppt]

## Slide 1
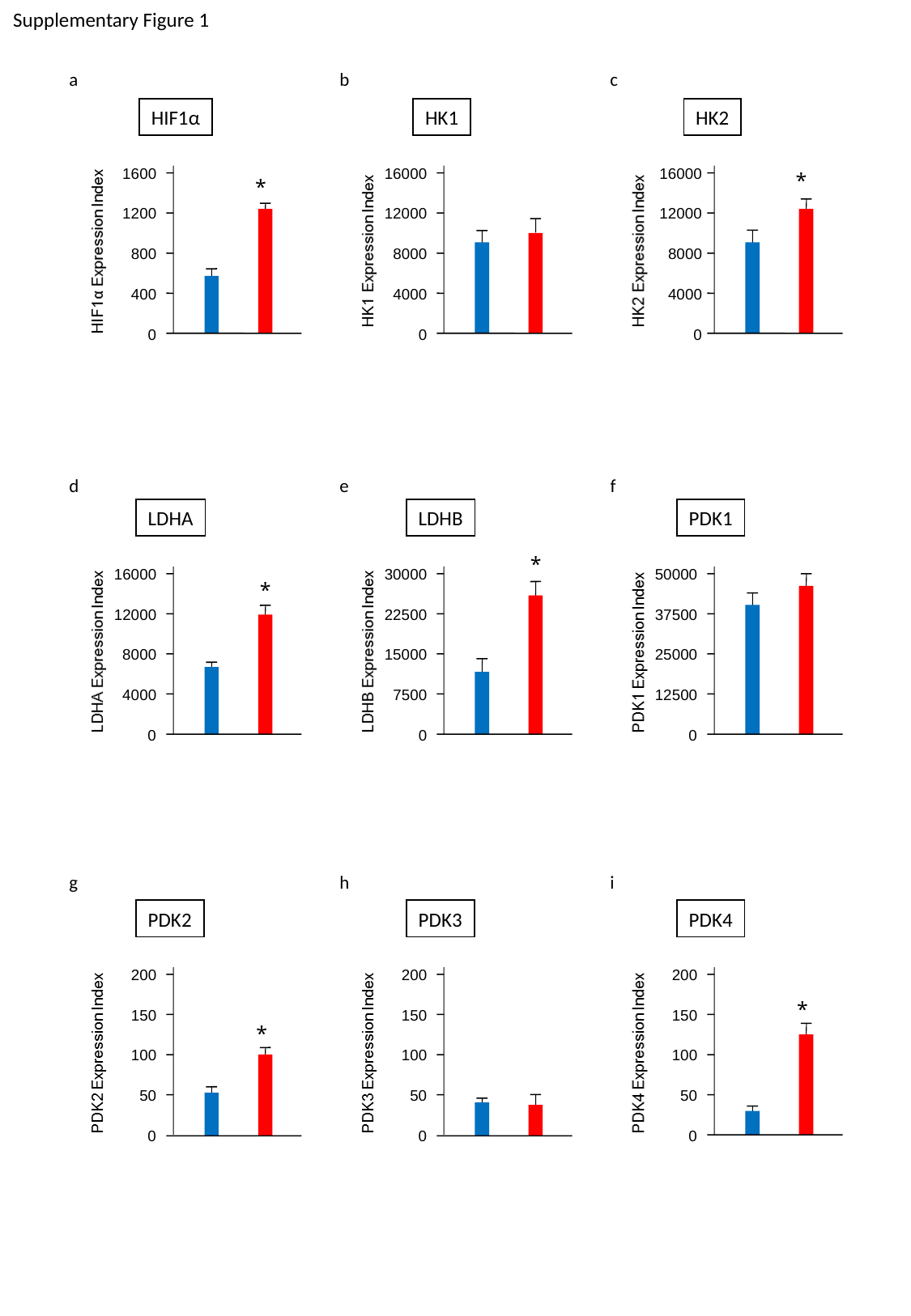

Supplementary Figure 1
a
b
c
HIF1α
HK1
HK2
1600
16000
16000
*
*
1200
12000
12000
800
8000
8000
400
4000
4000
0
0
0
d
e
f
LDHA
LDHB
PDK1
*
16000
30000
50000
*
12000
22500
37500
8000
15000
25000
4000
7500
12500
0
0
0
g
h
i
PDK2
PDK3
PDK4
200
200
200
*
150
150
150
*
100
100
100
50
50
50
0
0
0

## Slide 2
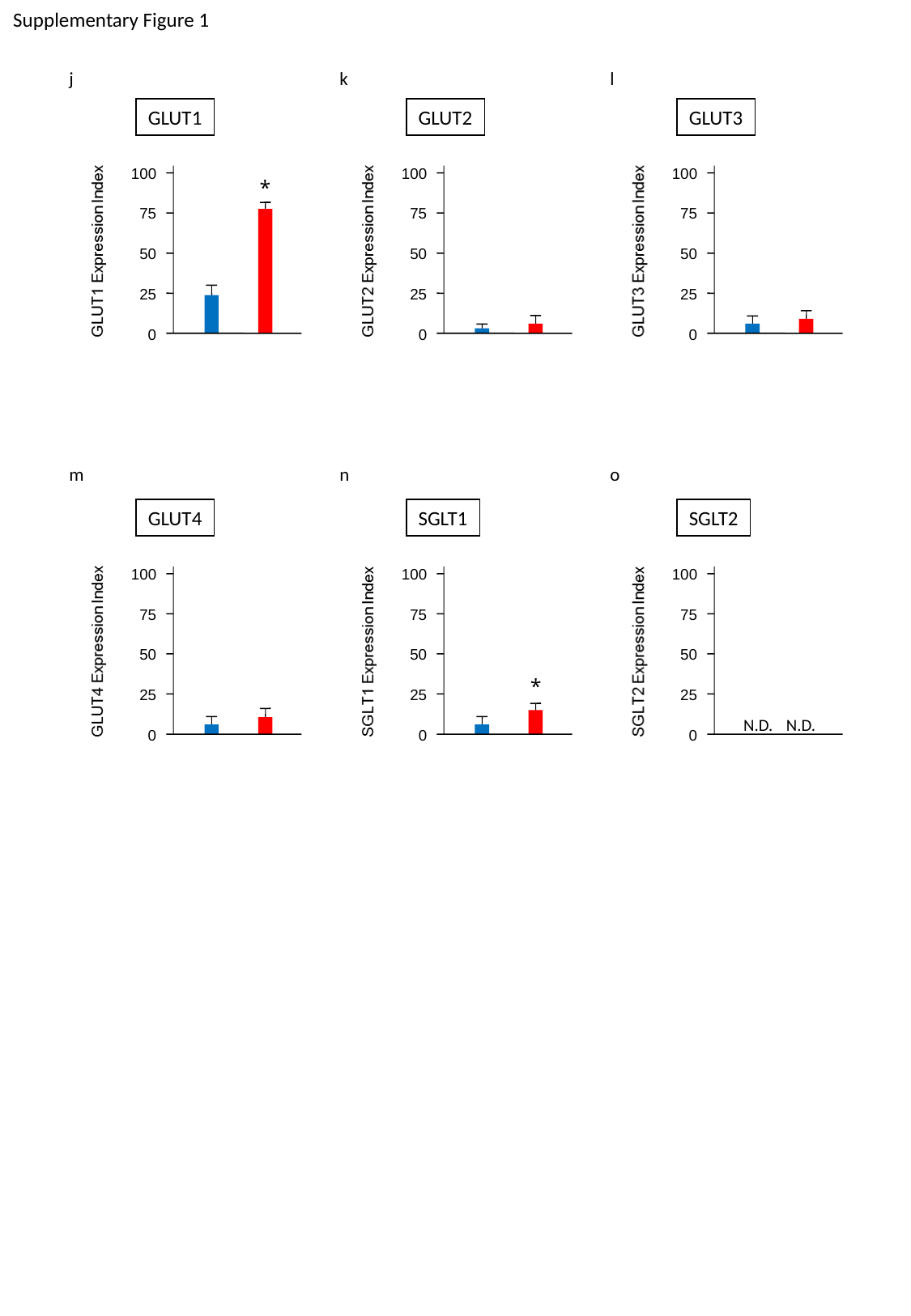

Supplementary Figure 1
j
k
l
GLUT1
GLUT2
GLUT3
100
100
100
*
75
75
75
50
50
50
25
25
25
0
0
0
m
n
o
GLUT4
SGLT1
SGLT2
100
100
100
75
75
75
50
50
50
*
25
25
25
N.D.
N.D.
0
0
0

## Slide 3
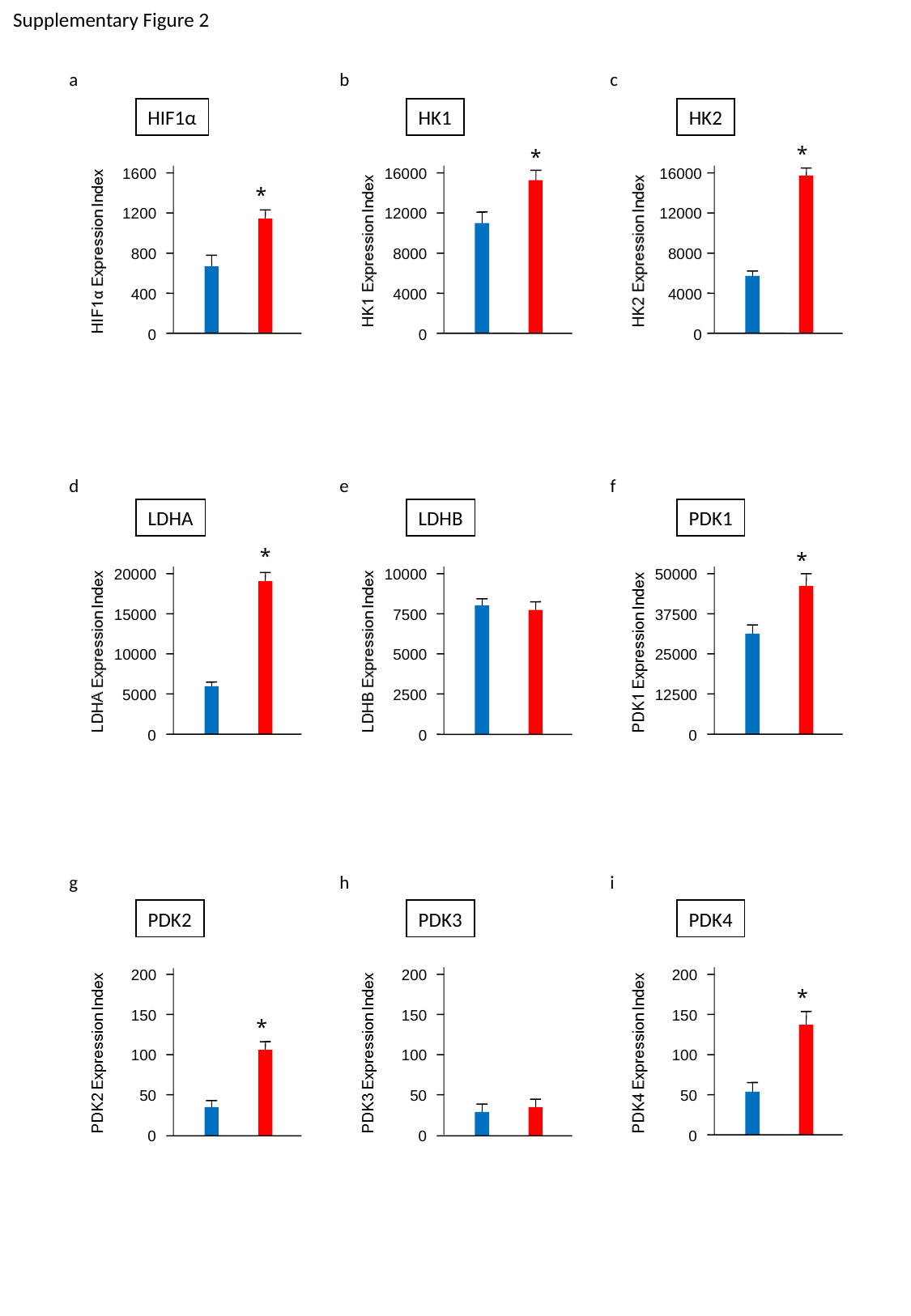

Supplementary Figure 2
a
b
c
HIF1α
HK1
HK2
*
*
1600
16000
16000
*
1200
12000
12000
800
8000
8000
400
4000
4000
0
0
0
d
e
f
LDHA
LDHB
PDK1
*
*
20000
10000
50000
15000
7500
37500
10000
5000
25000
5000
2500
12500
0
0
0
g
h
i
PDK2
PDK3
PDK4
200
200
200
*
150
150
150
*
100
100
100
50
50
50
0
0
0

## Slide 4
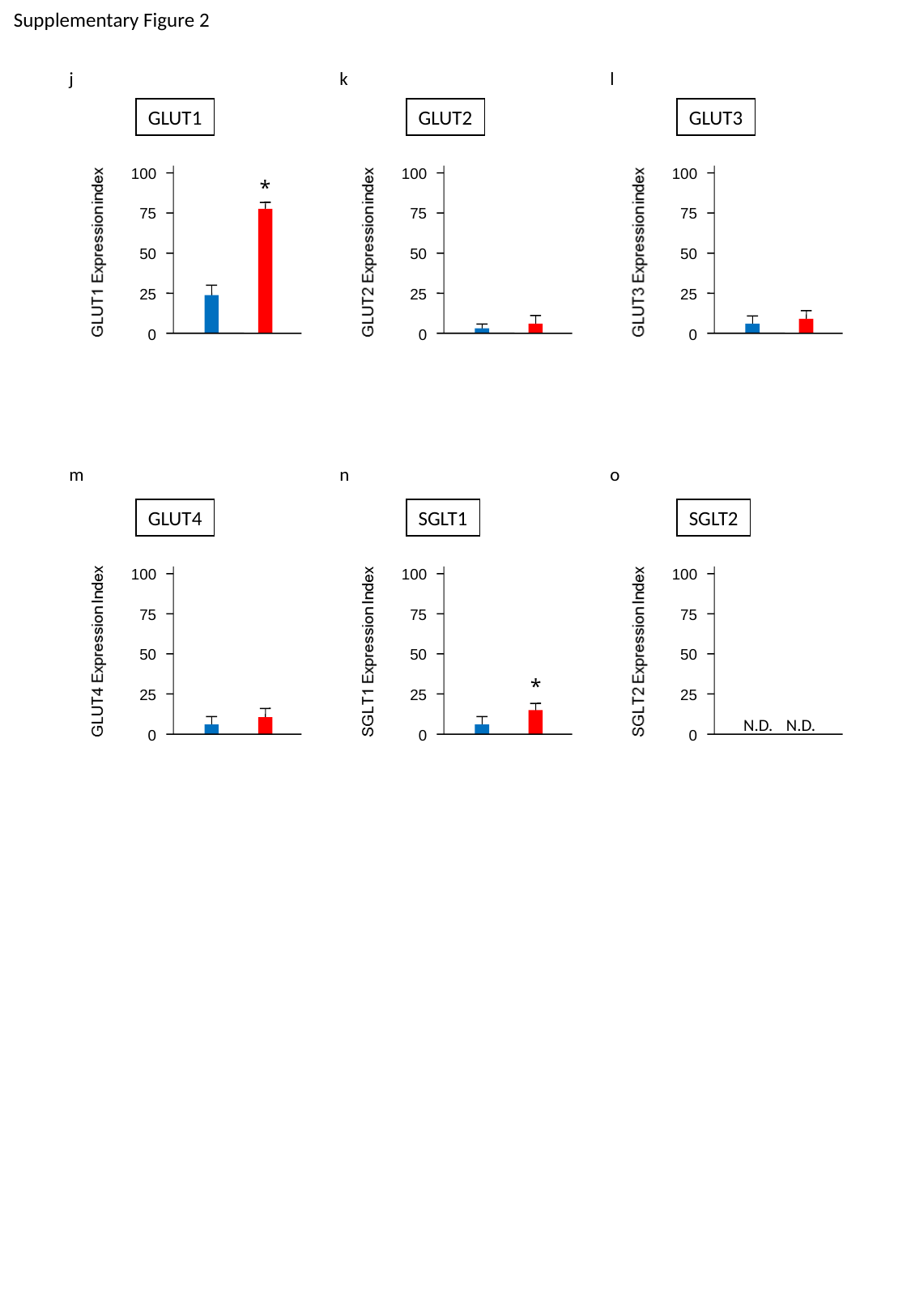

Supplementary Figure 2
j
k
l
GLUT1
GLUT2
GLUT3
100
100
100
*
75
75
75
50
50
50
25
25
25
0
0
0
m
n
o
GLUT4
SGLT1
SGLT2
100
100
100
75
75
75
50
50
50
*
25
25
25
N.D.
N.D.
0
0
0

## Slide 5
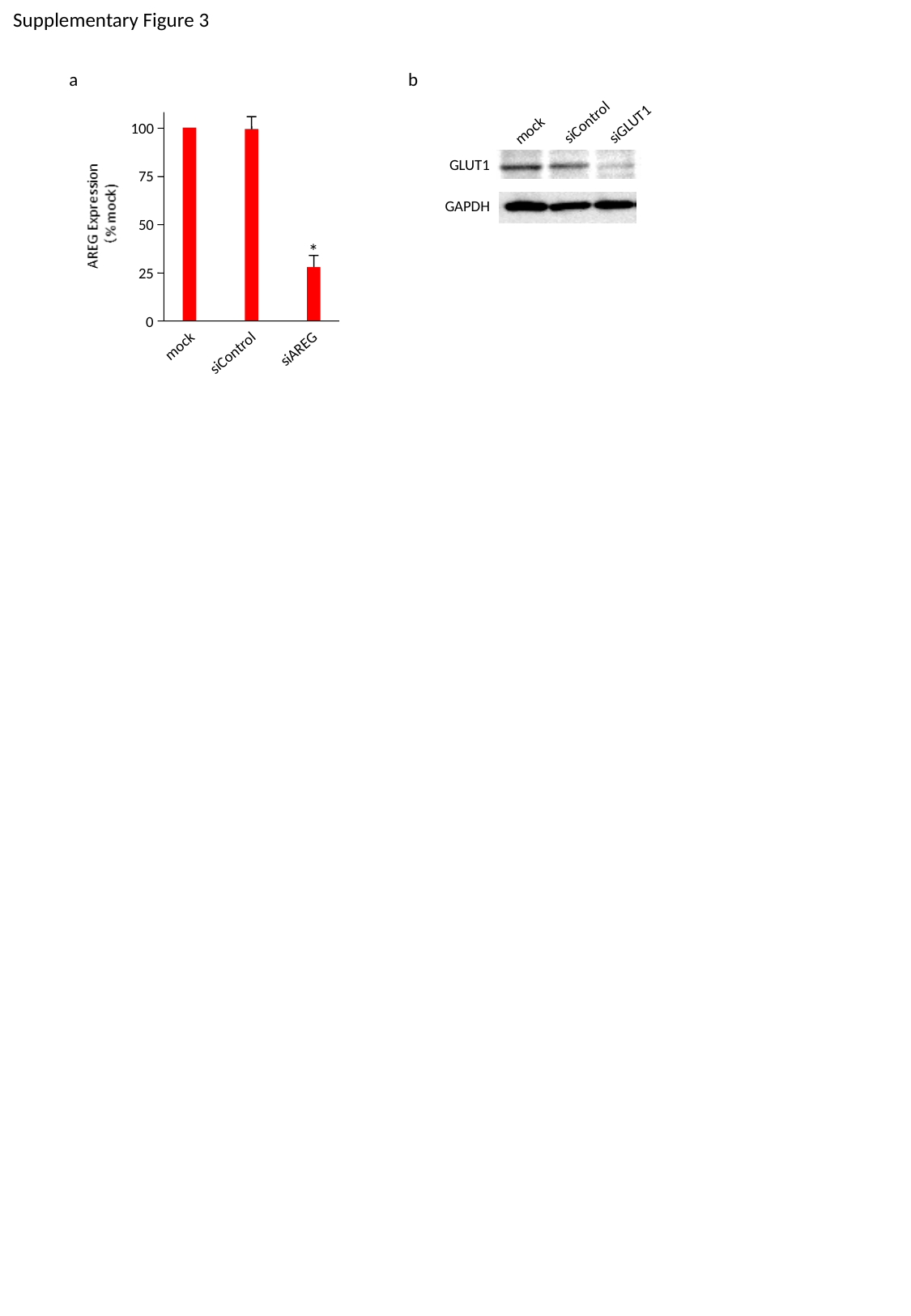

Supplementary Figure 3
a
b
siControl
siGLUT1
100
mock
GLUT1
75
GAPDH
50
*
25
0
mock
siAREG
siControl

## Slide 6
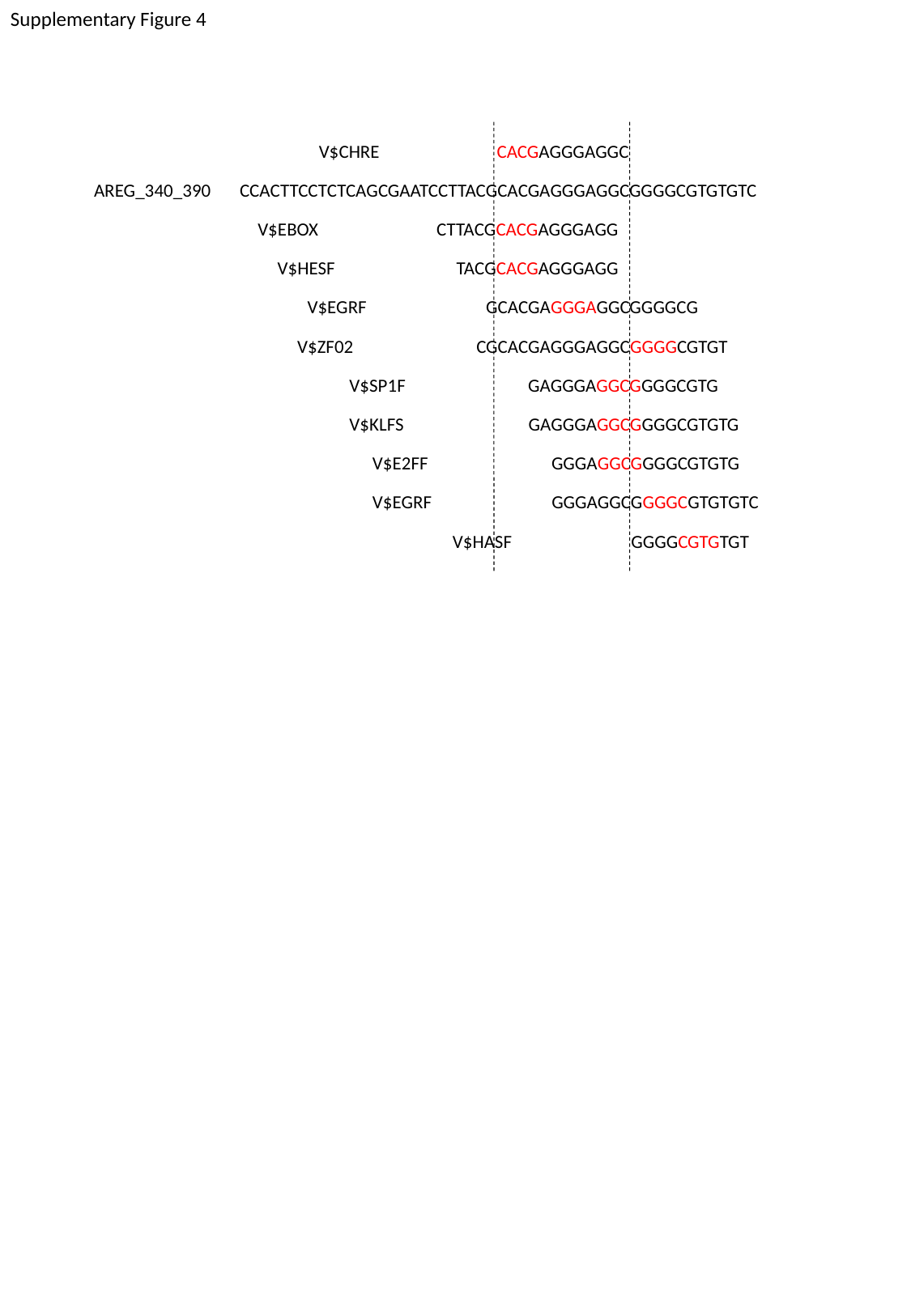

Supplementary Figure 4
V$CHRE
CACGAGGGAGGC
AREG_340_390
CCACTTCCTCTCAGCGAATCCTTACGCACGAGGGAGGCGGGGCGTGTGTC
V$EBOX
CTTACGCACGAGGGAGG
V$HESF
TACGCACGAGGGAGG
V$EGRF
GCACGAGGGAGGCGGGGCG
V$ZF02
CGCACGAGGGAGGCGGGGCGTGT
V$SP1F
GAGGGAGGCGGGGCGTG
V$KLFS
GAGGGAGGCGGGGCGTGTG
V$E2FF
GGGAGGCGGGGCGTGTG
V$EGRF
GGGAGGCGGGGCGTGTGTC
V$HASF
GGGGCGTGTGT
