# Supplementary material for: Warburg effect regulated by amphiregulin in the development of colorectal cancer
Source: Cancer Med. 2015 Jan 30;4(4):575–87. doi: 10.1002/cam4.416 (PMC4402072; doi:10.1002/cam4.416)
Supplement: Data S1 — Supplementary Materials and Methods. Figure S1. Alterations in the expression of genes involved in the Warburg effect in 2DC (blue bars) compared with 3DC (red bars) in HCT116 cells. (A) HIF-1α, (B) HK1, (C) HK2, (D) LDH-A, (E) LDH-B, (F) PDK1, (G) PDK2, (H) PDK3, (I) PDK4, (J) GLUT1, (K) GLUT2, (L) GLUT3, (M) GLUT4, (N) SGLT1, and (O) SGLT2. Data were measured in triplicate and represent mean ± SD. *P < 0.05 versus 2DC. Figure S2. Alterations in the expression of genes involved in the Warburg effect in 2DC (blue bars) and 3DC (red bars) in HT29 cells. (A) HIF-1α, (B) HK1, (C) HK2, (D) LDH-A, (E) LDH-B, (F) PDK1, (G) PDK2, (H) PDK3, (I) PDK4, (J) GLUT1, (K) GLUT2, (L) GLUT3, (M) GLUT4, (N) SGLT1, and (O) SGLT2. Data were measured in triplicate and represent mean ± SD. *P < 0.05 versus 2DC. Figure S3. Protein levels of AREG or GLUT1 under 3DC for 48 h after transfection with gene specific siRNAs into HCT116 cells. (A) The levels of AREG proteins in culture media of HCT116 cells were measured by ELISA. *P < 0.05. (B) Total GLUT1 proteins in HCT116 cells were detected by immunoblotting. GAPDH was used as internal control. Representative data of three independent experiments are shown. Figure S4. The primary structure of the −180/−130 promoter region of AREG. In silico analysis reveals the putative molecules bound to the promoter regions and their sequences (highlighted in red). Table S1. Forward and reverse primers of genes for real-time PCR amplification used in this study. Table S2. Primers used in this study. [file cam40004-0575-sd2.doc]

**Supplementary text**

**Title: Warburg effect regulated by amphiregulin in the development of colorectal cancer**

Sung Ouk Nam^1, 2^, Fusanori Yotsumoto^2, 3^, Kohei Miyata^1, 2^, Satoshi Fukagawa^1, 2^, Hiromi Yamada^2, 3^, Masahide Kuroki^2, 3^, Shingo Miyamoto^1, 2^

^1^Department of Obstetrics and Gynecology, Faculty of Medicine, Fukuoka University, Fukuoka, Japan; ^2^Central Research Institute for Advanced Molecular Medicine, Fukuoka University, Fukuoka, Japan; and ^3^Department of Biochemistry, Faculty of Medicine, Fukuoka University, Fukuoka, Japan

Correspondence to: Shingo Miyamoto, Department of Obstetrics and Gynecology, Faculty of Medicine, Fukuoka University, 7-45-1 Nanakuma, Jonan-ku, Fukuoka 814-0180, Japan, Tel: +81-92-801-1011, Fax: +81-92-865-4114, E-mail: [smiya@cis.fukuoka-u.ac.jp](mailto:smiya@cis.fukuoka-u.ac.jp)

E-mail address

Sung Ouk Nam; [nam100021@outlook.com](mailto:nam100021@outlook.com)

Yotsumoto Fusanori; [yotsumoto@cis.fukuoka-u.ac.jp](mailto:yotsumoto@cis.fukuoka-u.ac.jp)

Kohei Miyata; [kmiyata@outlook.com](mailto:kmiyata@outlook.com)

Satoshi Fukagawa; [md140020@cis.fukuoka-u.ac.jp](mailto:md140020@cis.fukuoka-u.ac.jp)

Hiromi Yamada; [hiromi@minf.med.fukuoka-u.ac.jp](mailto:hiromi@minf.med.fukuoka-u.ac.jp)

Kuroki Masahide; [kurokima@fukuoka-u.ac.jp](mailto:kurokima@fukuoka-u.ac.jp)

Shingo Miyamoto; [smiya@cis.fukuoka-u.ac.jp](mailto:smiya@cis.fukuoka-u.ac.jp)

**Supplementary Materials and Methods**

**Immunoblot analysis**

To detect GLUT1 proteins, HCT116 cells were then rinsed with PBS containing 1 mM sodium orthovanadate and lysed with 500 μl of RIPA buffer to obtain total cellular protein. Cell lysates were centrifuged at 15,000 × *g* for 15 min at 4°C. The supernatant was boiled for 5 min at 95 °C with 250 μl of 3× Laemmli sample buffer. All samples were subjected to sodium dodecyl sulfate-polyacrylamide gel electrophoresis and immunoblot analyses as described previously (1). Rabbit polyclonal anti-GLUT1 (ab652) was purchased from abcam (Abcam, Cambridge, UK). Peroxidase-conjugated anti-rabbit IgG were obtained from Zymed (San Francisco, CA).

**Supplementary References**

1. Miyamoto S, Hirata M, Yamazaki A, Kageyama T, Hasuwa H, Mizushima H, Tanaka Y, Yagi H, Sonoda K, Kai M, Kanoh H, Nakano H, Mekada E. 2004. Heparin-binding EGF-like growth factor is a promising target for ovarian cancer therapy. *Cancer Res* 64:5720–27.

**Supplementary Figure Legends**

**Supplementary Figure 1.** Alterations in the expression of genes involved in the Warburg effect in 2DC (blue bars) compared with 3DC (red bars) in HCT116 cells. (a) HIF-1α, (b) HK1, (c) HK2, (d) LDHA, (e) LDHB, (f) PDK1, (g) PDK2, (h) PDK3, (i) PDK4, (j) GLUT1, (k) GLUT2, (l) GLUT3, (m) GLUT4, (n) SGLT1, and (o) SGLT2. Data were measured in triplicate and represent mean ± SD. *P < 0.05 vs. 2DC.

**Supplementary Figure 2.** Alterations in the expression of genes involved in the Warburg effect in 2DC (blue bars) and 3DC (red bars) in HT29 cells. (a) HIF-1α, (b) HK1, (c) HK2, (d) LDHA, (e) LDHB, (f) PDK1, (g) PDK2, (h) PDK3, (i) PDK4, (j) GLUT1, (k) GLUT2, (l) GLUT3, (m) GLUT4, (n) SGLT1, and (o) SGLT2. Data were measured in triplicate and represent mean ± SD. *P < 0.05 vs. 2DC.

**Supplementary Figure 3. Protein levels of AREG or GLUT1 under 3DC for 48 h after transfection with gene specific siRNAs into HCT116 cells.** (A) The levels of AREG proteins in culture media of HCT116 cells were measured by ELISA. *P < 0.05 (B) Total GLUT1 proteins in HCT116 cells were detected by immunoblotting. GAPDH was used as internal control. Representative data of three independent experiments is shown.

**Supplementary Figure 4.** The primary structure of the −180/−130 promoter region of AREG. *In silico* analysis reveals the putative molecules bound to the promoter regions and their sequences (highlighted in red).

Table S1. Forward and reverse primers of genes for real-time PCR amplification used in this study

ATCTcattgccacgcgcccc

LDHA qPCR Forward primer

tcccagcctttcccccatcagg

LDHA qPCR Reverse primer

tgtagtggtaactgcaggagtccg

LDHB qPCR Forward primer

tggcagctgctgggatgaatgc

LDHB qPCR Reverse primer

TGGCCTATTACTTCACGGAGC

HKII qPCR Forward primer

GGAATGGACCTTACGAATGTTGG

HKI qPCR Reverse primer

TTTGACCACATTGCCGAATGC

HKI qPCR Forward primer

GGTCCATGAGACCAGGAAACT

HKII qPCR Reverse primer

GATGTGAATGGGCAGTTAGTC

PDK1 qPCR Forward primer

ATTCTCCCACCCATCAAGG

PDK1 qPCR Reverse primer

AGCCCATAACCAAAGCCAG

PDK2 qPCR Forward primer

AAGGAATAGTGGGTTAGGTGAG

PDK2 qPCR Reverse primer

TTAATAAGTCGCATGGCGC

PDK3 qPCR Forward primer

TGAAGCATCCCTGGGTTCAC

PDK3 qPCR Reverse primer

AACACCAGGAAAATCAGCC

PDK4 qPCR Forward primer

AAAACCAGCCAAAGGAGC

PDK4 qPCR Reverse primer

Table S2. Primers used in this study

AAGCTTTGGTCCTTCGCAGCGGC

Human AREG 3′ qPCR primer

GCTAGCCGCTGACCTTTTTATCTTGGG

Human AREG 5′ (−840) qPCR primer

GCTAGCATCGGCTGTGAGATGGTGTAG

Human AREG 5′ (−680) qPCR primer

GCTAGCATCAGGCAAAGTCACTCTTGG

Human AREG 5′ (−380) qPCR primer

GCTAGCCACTTCCTCTCAGCGAATC

Human AREG 3′ (−180) qPCR primer

GCTAGCCCGGCTGAGCCTATAAAGCG

Human AREG 5′ (−40) qPCR primer

ATATAAGCTTAACTTAAACCTCTAGCTGCA

Human AREG 5′ (−380) qPCR primer

ATATAAGCTTGACACACGCCCCGCCTCCCT

Human AREG 3′ (−130) qPCR primer

ATATAAGCTTTGGAGGCAGGCGGCGCCCCA

Human AREG 3′ (−80) qPCR primer

ATATAAGCTTGAGGGGGCCGCAGCCCATGA

Human AREG 3′ (−40) qPCR primer

ATATGCTAGCCCCACGGCCGGGCCTTGACGT

Human AREG 5′ (−80) qPCR primer

ATATGCTAGCCTCCGCGCGTGGTTTTCGGGTA

Human AREG 5′ (−130) qPCR primer

GTGTGTCCTCCGCGCGTGGT

Human AREG 5′ Inverse PCR primer

CGTAAGGATTCGCTGAGAGG

Human AREG 3′ Inverse PCR primer

TCCTGCTCGCCCTCAAAAAC

Human AREG 5′ ChIP primer

AAGGTGCTACCCGAAAACCA

Human AREG 3′ ChIP primer
